# A Plea for the Unification of Medical Societies1President’s address, Alumni Association of the Medical Department of the University of Buffalo, April 25, 1899.

**Published:** 1899-07

**Authors:** Z. J. Lusk

**Affiliations:** Warsaw, N. Y.


### BUFFALO MEDICAL JOURNAL.

VOL. XXXVIII. JULY, 1899. NO. 12.

### Original Communications.

### A PLEA FOR THE UNIFICATION OF MEDICAL SOCIETIES.[1]

By Z. J. LUSK, M. D., Warsaw, N. Y.

WHEN we met at the annual meeting one year ago, our country was on the threshold of war with a foreign nation. War with Spain was the subject of constant discussion. Little else occupied the minds of the people, indeed, the very atmosphere was laden with a belligerent odor.

We are again assembled at our annual reunion. In the meantime what wonderful changes have been wrought in the history of our country. On sea and land we have demonstrated to the world the metal of which we are composed. While we applaud the wonderful achievements of the navy and praise the valor of the army, little is known of the great work accomplished by the American surgeon, and of his patient, self-sacrificing activity upon the field, exposed to the deadly missiles of the enemy. We read of the tired soldier dropping into the trenches when darkness has covered the bloody battle-field, to gather rest for the day to follow, but little mention is made of the faithful surgeon who continues by the aid of the ''midnight oil'' the work of alleviating the suffering of the wounded and dying.

We read of the debates in congress relating to the promotions in the army and navy, and of the difficulties besetting the administration in properly distributing those honors. Let me ask, who has heard of any cases of insomnia on account of properly awarding the surgeon in recognition of his skilful work and bravery ? As surgeons we can appreciate the stupendous work and great responsibilities

1. President's address, Alumni Association of the Medical Department of the University of Buffalo, April 25, 1899.

of our modest, unassuming and capable Surgeon-general Sternberg. We understand that nothing in history compares with the marvelous recoveries from wounds and injuries (heretofore considered fatal) under the skill of the masterful Senn, and other distinguished surgeons. Then why is it that such slight recognition is accorded to that department?

It has been said that "when a person failed in everything else, he could, with little time and study, become a doctor." Hence the too common notion prevails that the medical profession does not contain the highest type of intellect as compared with scientific workers in other special lines.

This idea is based on very insufficient appreciation of the facts, as we are well aware that the medical profession throughout the world has its full share of the greatest minds as it has always had, and the point is, why should the contrary idea exist to any extent whatever among the average intellects of the general public?

I consider the answer to the foregoing of great importance to the profession and shall occupy a few moments in an effort to demonstrate wherein the fault principally lies.

A thorough investigation of the subject has persuaded me to the belief that the position we occupy is largely due, yes, almost entirely due to ourselves, and that the situation will remain unchanged until we adopt principles and measures in keeping with good, sound, common, business sense; indeed, commensurate with the advancement and standard governing the closing years of this century instead of adhering to those prevailing one hundred years ago.

In the first place, we sadly ignore the generally accepted axiom that "in union there is strength," also to that other truism, that "united we stand, divided we fall," and are so divided and subdivided again into so-called medical associations and societies, national and state, that whereas we should, and would, if united, possess a powerful influence politically and socially, as at present organised we exercise very little, and deserve less.

There are in the United States nearly one hundred thousand members of the regular profession. Imagine our power socially and politically if we paid allegiance to one national association, for instance, the one which has always taken the lead in power and numbers, viz.: The American Medical Association. This should be strengthened by an enormously increased membership to which every physician should feel the same loyalty as to the country in which he lives. Although the *Journal of the American Medical*

*Association* is one of the best, if not the best journal published in this country today, being exclusively the property of the members of the profession, imagine what it would be with the support following a united profession.

Permit me to mention a few of the twenty-eight national medical associations into which the profession of the United States is divided : The American Medical Association; The Association of American Physicians; The American Academy of Medicine; The National Association of Railway Surgeons; The American Academy Railway Surgeons; The American Association of Obstetricians and Gynecologists; The American Gynecological Society; The Southern Surgical and Gynecological Association; The American Laryngological, Rhinological and Otological Society; The American Laryngological Association, and so on *ad infinitum.*

Then in our own State of New York there is The New York State Medical Association; The Medical Society of the State of New York; The New York State Association of Railway Surgeons, and so forth.

Now why could not a majority of these numerous societies accomplish fully as important results in their special lines organised in sections, thus representing branches of the one great body? Why is this marked isolation necessary? I will leave the answer with you.

Another idiosyncrasy demanding reform is the organisation of innumerable so-called medical colleges and post-graduate medical schools. The craze of adding professor to their cognomen seems to afflict especially the profession of the great Mississippi Valley.

In the State of Missouri[1] there has been organised since 1840, thirty-three medical colleges; seventeen have passed away, leaving sixteen in full operation in 1895.

The following is taken from the annual address of Dr. Richmond, of St. Joseph, President of the Missouri State Medical Society :

A few, shrewd and ambitious for self-promotion, seeing how they would reap advantages from a professorship, organised a college. Those who were left out, not willing to be overtopped by the big professors, organised a second. Still there were a few who were not supplied and disaffection springing up in one, it swarmed and a third was the result. Of the forty-four doctors then in town I was one of the four who was not a professor, a distinction of which I am not ashamed. In selecting their faculties it was not necessary to go beyond the city limits, only to the suburbs.

These numerous schools of medicine in their effort to obtain clinical material, have lowered the standing of the profession by freely supplying medical aid to those abundantly able to pay, creating the impression among the general public that professional services should be obtained with slight expense. This idea is also emphasised by the careless unbusinesslike physician who has the unenviable reputation of never collecting nor paying his bills.

The sentimental nonsense of which we read of the so-called kind old pauper, "Weelum MacClure," should not belong to the present age. While the physician willingly does more charity work than those belonging to any other profession, he should draw the line between the poor patient and the one able to pay for services rendered, and I can assure you that nothing detracts from professional honor more in the eyes of the public, no, nor even in that class who can pay and won't, unless obliged to, than the physician who continues to respond to calls without even making an effort to secure adequate return.

I have heard patients who were always very exacting about prompt response to their calls, say, "Oh! we always pay the doctor the last thing." Such families should get very little rest after a reasonable time, unless payment is made. Should you lose their clientage, you have suffered no loss.

Another subject which should engage the serious consideration of the profession, is the part we take in the politics of our country. While in the past we have been admonished to avoid participation in political affairs, the time has arrived when it is absolutely essential that we should make our power known, not necessarily in seeking official positions, but in the selecting of those who will recognise our position and our reasonable demands.

We leave[2] all the law-making to the politicians, most of whom care nothing about us. We allow ourselves to be duped and imposed upon on every side with the most idiotic complacency. The fact of the matter is, we have nobody to blame but ourselves. And another truth is that we can right these wrongs just as soon as we will do so and pull together for that purpose.

"I am aware that there are those in our profession who shrink at the thought of combining medicine and politics, regarding them as entirely incompatible and exceedingly nauseating. But there never was a time in the history of medicine when such a mixture was more urgently needed than the present, and, instead of attempting so much unappreciated gratuitous service to the public, let us recognise some of our own needs and spend at least some time in doctoring ourselves, for, in the language of Mr. Cleveland, "Never did patient need your medical treatment more than the body politic now needs the watchful care of your patriotic and disinterested citizenship."

If laws[3] were needed to abolish abuses which your professional investigations have unearthed, your fraternity should not be strangers to the agencies which make the laws.

If enactments already in force are badly neglected or badly executed, you should not forget that it is your privilege and duty to insist upon their rigorous and honest enforcement.

Let us remind you of the application, to your case, of the truth embodied in the homely injunction, "If you want a job done well do it yourself."

No object or personal ambition and no activity of professional life should be permitted to withhold from our government the tithe of devotion and service due from its thoughtful, intelligent and educated citizens.

Beyond all questions[4] things have come to such a pass that for medical men, matters pertaining to professional, and sanitary welfare must henceforth outweigh all considerations of partisanship.

It is a shame for medical men to sell themselves in future for the advantage of politicians and commercial interests while the far higher and more vitally important question of scientific advance and the cure of disease are utterly neglected. It is our inescapable duty henceforth to judge of men and parties from the professional standpoint, and to make our power felt with administrators and legislators for purposes other than those represented by lobbies and bosses.

I believe the profession is awakening to the demands of the times. That we are beginning to move in the right direction.

The following item appeared in a recent journal:

The two oldest medical colleges[5] in St. Louis are proposing to amalgamate, viz: The Missouri Medical College and the St. Louis College of Medicine.

In this connection let us congratulate ourselves upon the union of the forces of the Niagara Medical College with the Medical Department of the University of Buffalo. That with the reception of their alumni into full membership into our association, marks an historical epoch to which we can always point with pride.

Let us not forget our allegiance to our Alma Mater. Let us feel it an honor that we are members of her large and growing family. And, finally, whatever our relation to other medical societies, let us not forget our fraternal interest in our Alumni Association.

BIBLIOGRAPHY.

1. Medical Mirror, Vol. VI., page 258.
2. Journal of the American Medical Association, Vol. XXIX., page 389.
3. Ibid., Vol. XXVIII., page 269.
4. Ex-President Cleveland's address at the New York Academy of Medicine.
5. Ibid., Vol. XXVIII., page 86.

